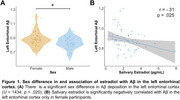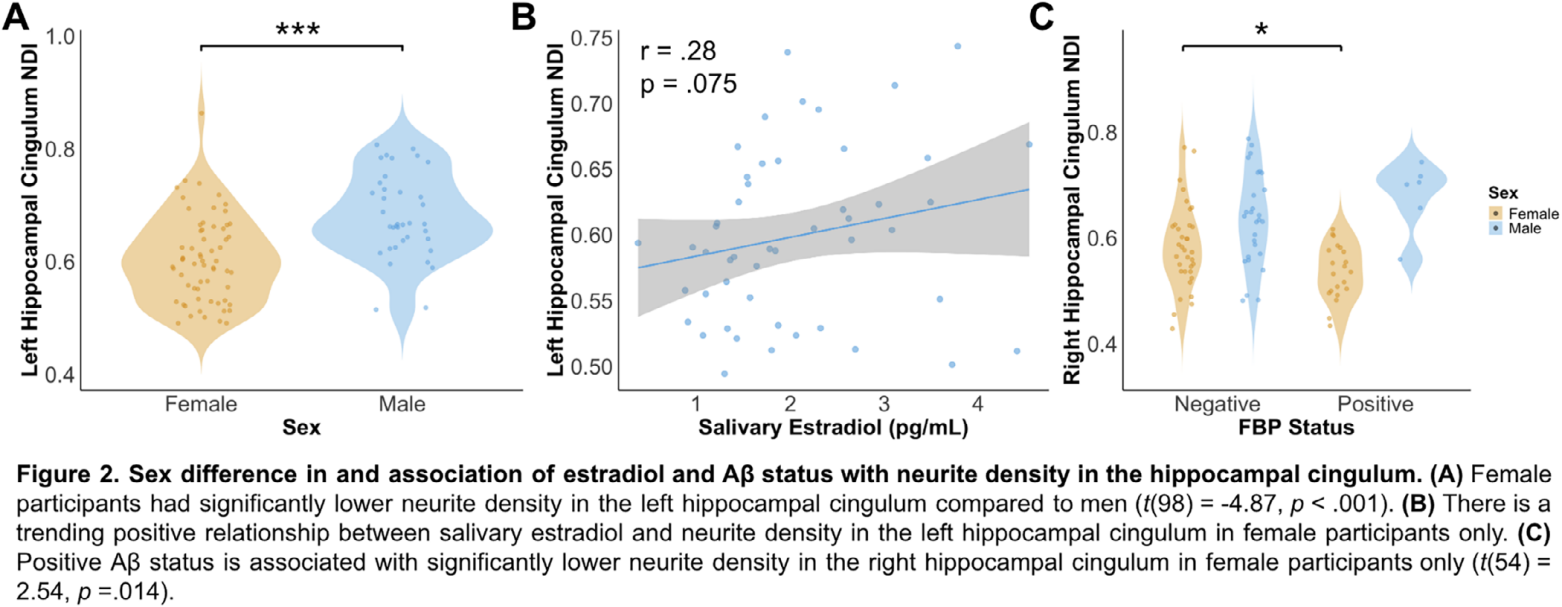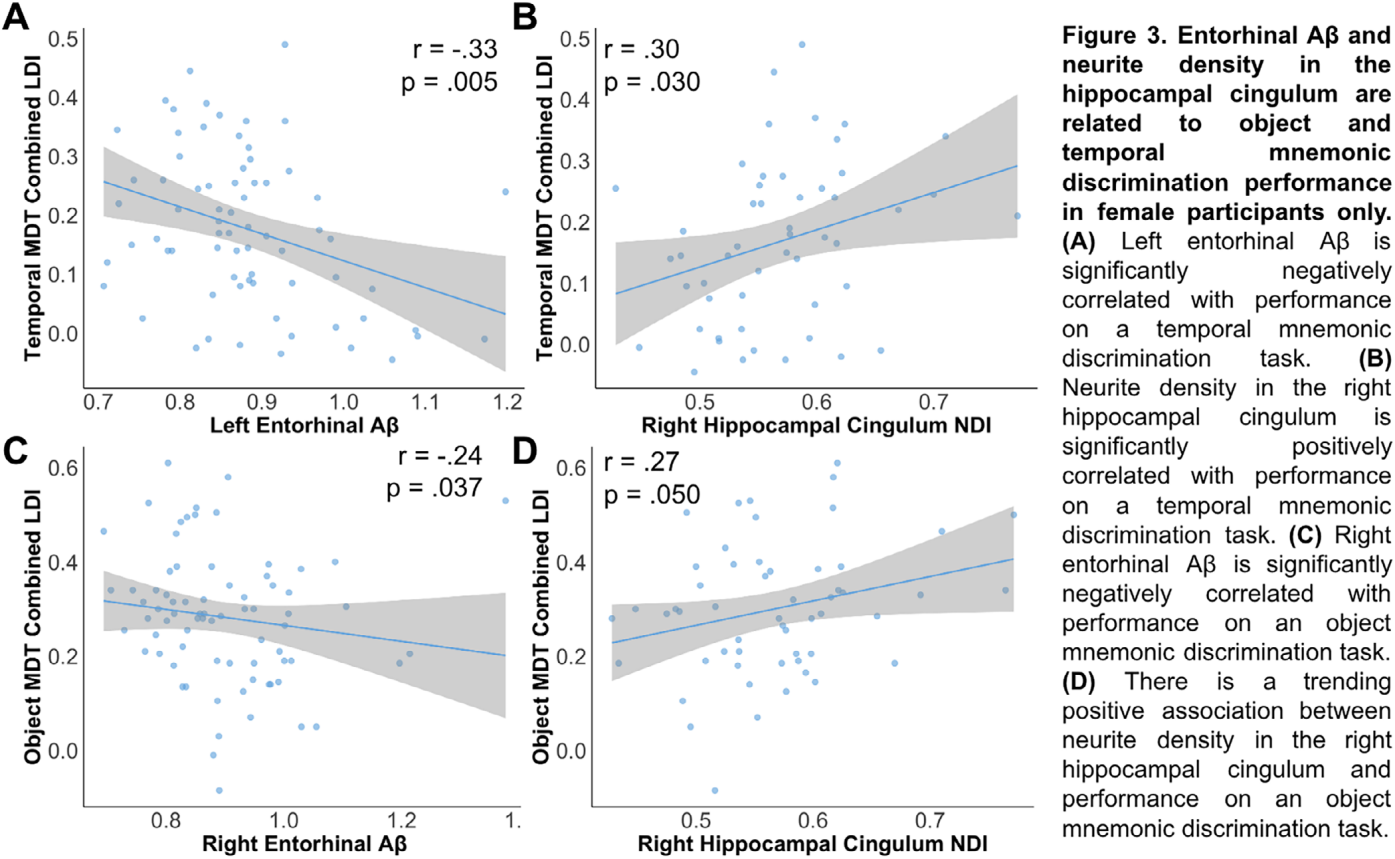# Sex‐specific effects of estradiol on regional amyloid‐β, white matter integrity, and hippocampal‐dependent memory in cognitively unimpaired older adults

**DOI:** 10.1002/alz70861_108819

**Published:** 2025-12-23

**Authors:** Emily Yi, Dana M Parker, Lisa M. Taylor, Soyun Kim, Elizabeth A. Thomas, Michael A Yassa, Jenna N. Adams

**Affiliations:** ^1^ University of California, Irvine, Irvine, CA USA

## Abstract

**Background:**

Evidence suggests menopause‐driven estradiol withdrawal increases vulnerability to AD pathogenesis in women. While studies have shown women and men accumulate global amyloid‐β (Aβ) at similar rates, the regional specificity of sex‐ and estradiol‐related Aβ has not been studied. Whether decreased estradiol renders medial temporal lobe (MTL) white matter more vulnerable in women and contributes to related memory deficits has not been investigated. We examined estradiol’s relation to sex‐specific regional Aβ deposition, MTL white matter integrity, and hippocampal‐dependent memory performance in cognitively unimpaired (CU) postmenopausal women and older men.

**Method:**

We analyzed 129 CU adults (64% female; 60‐87 years) from the Biomarker Exploration in Aging, Cognition and Neurodegeneration (BEACoN) study. Salivary estradiol was measured using a saliva‐optimized immunoassay in a subset of 87 participants (72% female). Aβ was measured with 18F‐florbetapir‐PET, and global Aβ, Aβ status, and regional Aβ in the entorhinal cortex (EC) and precuneus were quantified. White matter integrity of the hippocampal cingulum tract was measured with multishell diffusion MRI using neurite density index (NDI). Hippocampal‐dependent memory was measured using mnemonic discrimination tasks. Mann‐Whitney U tests assessed sex differences and Spearman’s rank correlations tested relationships between continuous variables.

**Result:**

No sex difference or relationship existed between salivary estradiol levels and global Aβ or Aβ status. However, regional analyses revealed a significant sex difference in EC Aβ and a negative correlation between estradiol levels and EC Aβ only in women (Figure 1). Women had significantly lower hippocampal cingulum NDI than men (Figure 2A). There was a trend towards a positive association between estradiol levels and hippocampal cingulum NDI only in women (Figure 2B). Women classified as Aβ+ had significantly lower hippocampal cingulum NDI compared to women who were Aβ‐ (Figure 2C). EC Aβ and hippocampal cingulum NDI were significantly correlated with object and temporal mnemonic discrimination performance in women, but not men (Figure 3).

**Conclusion:**

These findings suggest decreased estradiol in women over 60 is associated with increases in entorhinal Aβ accumulation and reduced MTL white matter integrity, which may confer sex‐specific deficits in hippocampal‐dependent memory. These biomarkers may reflect AD risk unique to women, with potential to help narrow the window for intervention.